# 通过式固相萃取-超高效液相色谱-串联质谱法测定沉积物中草铵膦、草甘膦及其代谢物

**DOI:** 10.3724/SP.J.1123.2024.03008

**Published:** 2025-02-08

**Authors:** Xiao YANG, Zhonggui XIE, Xiaoling LI, Wenwen SUO, Xiangyi CHEN, Yiwen WAN

**Affiliations:** 1.湖南省水产科学研究所,湖南长沙410153; 1. Hunan Fisheries Science Institute, Changsha 410153, China; 2.农业农村部渔业产品质量检验测试中心(长沙),湖南长沙410153; 2. Fishery Products Quality Testing Center, Ministry of Agriculture and Rural Affairs, Changsha 410153, China

**Keywords:** 超高效液相色谱-串联质谱, 通过式固相萃取, 草铵膦, 草甘膦, 代谢物, 沉积物, 非衍生化, ultra-high performance liquid chromatography-tandem mass spectrometry (UHPLC-MS/MS), pass-through solid-phase extraction, glufosinate, glyphosate, metabolites, sediment, non-derivatization

## Abstract

建立了通过式固相萃取-超高效液相色谱-串联质谱法测定沉积物中草铵膦、草甘膦及其6种代谢物(3-甲基磷酸亚基丙酸、*N*-乙酰草铵膦、氨甲基膦酸、*N*-乙酰氨甲基膦酸、*N*-乙酰草甘膦、*N*-甲基草甘膦)的方法。样品采用4%(体积分数)氨水溶液提取,提取液经PRiME HLB固相萃取柱净化,过0.22 μm聚醚砜滤膜后供超高效液相色谱-串联质谱测定。目标化合物使用Metrosep A Supp 5阴离子色谱柱(150 mm×4.0 mm, 5 μm)分离,以水和200 mmol/L碳酸氢铵溶液(含0.05%(v/v)氨水)作为流动相进行梯度洗脱,在电喷雾离子源(ESI)、负离子扫描和多反应监测(MRM)模式下进行测定,基质匹配外标法定量。结果表明,草铵膦、草甘膦及其代谢物在15 min内即可完成色谱分离,色谱峰形良好,响应值高,目标化合物在2.0~200.0 μg/L范围内线性关系良好,相关系数均大于0.995。草铵膦、3-甲基磷酸亚基丙酸、*N*-乙酰草铵膦、*N*-乙酰氨甲基膦酸、*N*-乙酰草甘膦、*N*-甲基草甘膦的检出限为5 μg/kg,定量限为20 μg/kg;草甘膦、氨甲基膦酸的检出限为10 μg/kg,定量限为30 μg/kg。以空白沉积物为基质样品,在3个加标水平(定量限、5倍定量限和10倍定量限)下,低有机质含量的沉积物中目标化合物的平均回收率为78.5%~107%,相对标准偏差为1.32%~14.7%(*n*=6);高有机质含量的沉积物中目标化合物的平均回收率为76.4%~113%,相对标准偏差为2.60%~11.2%(*n*=6)。采用本方法对池塘、湖泊、水库、河流等不同类型的沉积物样品进行测定,结果显示,湖泊、水库、河流沉积物样品中未检出草铵膦、草甘膦及其代谢物,1个池塘沉积物样品中检出草甘膦和氨甲基膦酸,检出含量分别为31.7 μg/kg和52.3 μg/kg。本研究建立的方法具有简单、快速、绿色环保、准确度和灵敏度高、重复性好等优势,适用于沉积物中草铵膦、草甘膦及其代谢物的快速检测,为研究其在沉积物中的残留特征和环境行为提供了技术支持。

## Abstract (en)

Glufosinate (GLUF) and glyphosate (GLY) are nonselective phosphorus-containing amino acid herbicides that are widely used in agricultural gardens and noncultivated areas. These herbicides give rise to a number of key metabolites, with 3-methyl phosphinicopropionic acid (MPPA), *N*-acetyl glufosinate (*N*-acetyl GLUF), aminomethyl phosphonic acid (AMPA), *N*-acetyl aminomethyl phosphonic acid (*N*-acetyl AMPA), *N*-acetyl glyphosate (*N*-acetyl GLY), *N*-methyl glyphosate (*N*-methyl GLY) as the major metabolites obtained from GLUF and GLY. Extensive use of these herbicides may lead to their increased presence in the environment, especially aquatic ecosystems. An increasing number of research studies into the toxicities of GLUF, GLY, and their metabolites have shown that these herbicides are potentially toxic to aquatic biota. GLUF and GLY, as well as their metabolites, are extremely polar and water-soluble, and they lack chromogenic and fluorescent groups; therefore, their concentrations are difficult to determine using conventional methods. Most analytical methods used to date have largely depended on derivatization procedures, leading to overall determination processes that are tedious and time-consuming. Therefore, establishing a quick and sensitive method that does not require derivatives for determining GLUF, GLY, and their metabolites in water environments, including surface water, sediments, and aquatic organisms, is an important endeavor.

In this study, a new approach was developed based on pass-through solid-phase extraction coupled with ultra-high performance liquid chromatography-tandem mass spectrometry (UHPLC-MS/MS) to determine GLUF, GLY, and their metabolites, including MPPA, *N*-acetyl GLUF, AMPA, *N*-acetyl AMPA, *N*-acetyl GLY, and *N*-methyl GLY, in sediments. Samples were extracted with 4% (v/v) ammonia water and purified using PRiME HLB pass-through solid-phase extraction columns. The extracts were filtered through a polyethersulfone microfiltration membrane and analyzed by UHPLC-MS/MS. Compounds were separated on a Metrosep A Supp 5 column (150 mm×4.0 mm, 5 μm) using gradient elution with water and 200 mmol/L ammonium hydrogen carbonate solution containing 0.05% (v/v) ammonia water as the mobile phases. Analytes were detected using MS/MS with a negative electrospray ionization (ESI^-^) source in the multiple reaction monitoring (MRM) mode. A matrix-matched external-standard approach was used for quantitative analysis. GLUF, GLY, and their metabolites were detected within 15 min with good peak shapes and high responses. Calibration curves were linear in the range of 2.0-200 μg/L, with correlation coefficients exceeding 0.995. This method delivered limits of detection (LODs) and limits of quantification (LOQs) of 5 μg/kg and 20 μg/kg, respectively, for GLUF, MPPA, *N*-acetyl-GLUF, *N*-acetyl AMPA, *N*-acetyl GLY, and *N*-methyl GLY, and 10 μg/kg and 30 μg/kg, for GLY and AMPA, respectively. Average spiked recoveries at three levels (LOQ, 5LOQ, 10LOQ) in sediment with low organic matter content were in the range of 78.5%-107%, and the relative standard deviations (RSDs) were in the range of 1.32%-14.7% (*n*=6). Average spiked recoveries of 76.4%-113% were determined for three levels (LOQ, 5LOQ, and 10LOQ) in sediments with high organic matter contents, with RSDs of 2.60%-11.2% (*n*=6). The developed method was used to analyze GLUF, GLY, and their metabolites in ponds, lakes, reservoirs, and river sediments. No target compounds were detected in any sediment sample obtained from a lake, reservoir, or river; however, GLY and AMPA were detected in one pond-sediment sample at levels of 31.7 and 52.3 μg/kg, respectively. The developed approach is simple, fast, and green; moreover, it offers advantages, including high accuracies, high sensitivities, and good reproducibilities. Accordingly, the developed method is suitable for determining GLUF, GLY, and their metabolites in sediments and can provide technical support for studying residue characteristics and environmental behavior in sediments.

草铵膦(GLUF)和草甘膦(GLY)是一类非选择性、灭生性除草剂,因其具有高效、廉价、低毒等特点,在全世界范围内广泛用于茶园、果园、非耕地等农用地的杂草控制^[1]^。GLUF和GLY分子结构上含有氨基、羧基和膦酸基,是极强的两性化合物,易发生降解和代谢。GLUF的主要代谢物为3-甲基磷酸亚基丙酸(MPPA)、*N*-乙酰草铵膦(*N*-acetyl GLUF), GLY的主要代谢物为氨甲基膦酸(AMPA)、*N*-乙酰氨甲基膦酸(*N*-acetyl AMPA)、*N*-乙酰草甘膦(*N*-acetyl GLY)、*N*-甲基草甘膦(*N*-methyl GLY),二者的代谢物化学性质与原药类似,具有有机膦化合物相似的毒性。GLUF和GLY在施用后可通过地表径流、雨水冲刷以及地下水渗透等方式进入水环境中^[2]^。残留于水环境中的农药在水、沉积物和水生动植物等不同生态位中进行迁移转化、富集和代谢,对于水生动物和人类健康带来潜在威胁与长期毒性^[2,3]^。为控制GLUF、GLY及其代谢物的污染,有必要建立沉积物中该类化合物的检测方法。

GLUF、GLY及其代谢物极性强,易溶于水,难溶于有机溶剂,在常规反向色谱柱上无保留,且缺少发色和荧光基团,采用常规检测技术手段难以对其直接定性定量分析测定^[4,5]^。目前,GLUF、GLY及其代谢物的测定方法主要有气相色谱法(GC)^[6]^、气相色谱-质谱法(GC-MS)^[7]^、高效液相色谱法(HPLC)^[8]^、离子色谱法(IC)^[9,10]^、高效液相色谱-串联质谱法(HPLC-MS/MS)^[11,12]^等。GC、GC-MS、HPLC需衍生化,前处理操作繁琐耗时,重现性差;IC灵敏度较低,基质干扰大,适应范围窄;HPLC-MS/MS具有灵敏度高、分析时间短、抗干扰能力强等优点,已发展成农药残留分析的常用技术,适合环境样品中农药残留的定性定量检测^[13]^,可开发出直接测定GLUF、GLY及其代谢物的非衍生化方法。沉积物基质较为复杂,含有有机质、腐殖酸、矿物质、重金属离子等成分,能与GLUF、GLY及其代谢物发生强烈的吸附和络合反应,影响待测物的提取效率且干扰其测定。检测沉积物中GLUF、GLY及其代谢物的关键是前处理过程必须采用恰当的提取和净化手段,以便提高提取效率并减少样品基质对待测物的干扰。目前,国内外针对GLUF、GLY、AMPA 3种除草剂检测的研究报道较多,测定对象主要为植物源性食品^[5,14⇓⇓-17]^、水^[18⇓-20]^、土壤^[21,22]^、生物体液^[23⇓-25]^等,样品前处理主要采用直接进样^[12,18]^、固相萃取^[5,19,21,23]^或分散固相萃取^[14⇓⇓-17]^等手段。直接进样检测操作虽简便快速,但一般只适用于基质成分较为简单的样品。传统的固相萃取操作繁琐耗时,易造成待测物的损失而影响定量的准确性。分散固相萃取是一种简单、高效、快速的前处理技术,但在实际应用中需要对净化吸附剂的种类和用量进行优化。PRiME HLB固相萃取柱是新一代的通过式固相萃取柱,具有水可浸润性和反相保留特性,使用时无需活化、淋洗和洗脱,可将提取液直接过柱以实现对复杂样品基质中酸性、碱性和中性化合物的反相净化^[26,27]^。目前,关于PriME HLB固相萃取净化-高效液相色谱-串联质谱法同时检测沉积物中GLUF、GLY及其6种代谢物(MPPA、*N*-acetyl GLUF、AMPA、*N*-acetyl AMPA、*N*-acetyl GLY、*N*-methyl GLY)的方法尚未见报道。为了便于系统研究GLUF、GLY及其代谢物在沉积物中的残留特征和生态风险,建立一套操作简单、灵敏度高、重复性好的快速检测方法显得十分必要。

本文采用PRiME HLB固相萃取净化,结合超高效液相色谱-串联质谱(UHPLC-MS/MS),建立了一种同时测定沉积物中GLUF、GLY、MPPA、*N*-acetyl GLUF、AMPA、*N*-acetyl AMPA、*N*-acetyl GLY、*N*-methyl GLY共8种化合物的方法。该方法前处理无需有机试剂和衍生化,操作简单快速,绿色环保,灵敏度高,回收率和重复性好,适用于大批量样品的快速检测,为研究GLUF、GLY及其代谢物在沉积物中的残留状况和环境行为提供可靠的技术支持。

## 1 实验部分

### 1.1 仪器与试剂

ExionLC AD/Triple Quad 5500+超高效液相色谱-串联质谱仪(美国SCIEX公司); SBAB-57265固相萃取装置(美国Supelco公司); KQ-500DE超声波清洗器(昆山市超声仪器有限公司); Multifuge X1R台式离心机、涡旋振荡器(美国Thermo Fisher Scientific公司); HXLG-12-50B立式冷冻干燥机(上海沪析实业有限公司); ZWM-UT1-20超纯水机(湖南中沃水务环保科技有限公司); Metrosep A Supp 5阴离子色谱柱(150 mm×4.0 mm, 5 μm;瑞士万通公司);十八烷基键合硅胶吸附剂(C_18_, 40~63 μm)、*N*-丙基乙二胺(PSA, 40~63 μm)、石墨化炭黑(GCB, 120~400目)(上海安谱实验科技有限公司); Oasis PRiME HLB固相萃取柱(3 mL, 60 mg;美国Waters公司)。

GLUF、GLY、MPPA、*N*-acetyl GLUF、AMPA、*N*-acetyl AMPA、*N*-acetyl GLY、*N*-methyl GLY(纯度>96%,德国Dr. Ehrenstorfer公司);碳酸氢铵(纯度>99.99%,上海阿拉丁生化科技股份有限公司);实验用水为超纯水(18.25 MΩ·cm)。

### 1.2 样品采集与制备

沉积物样品按照SC/T 9102.3-2007《渔业生态环境监测规范第3部分:淡水》规定的方法进行采集。沉积物样品采样量应不少于500 g,于-20 ℃预冷冻24 h,经冷冻干燥机(-50 ℃,真空度<20 Pa,冷冻干燥10 h)冻干,剔除石块和植物体等异物,用研钵研磨后过孔径0.25 mm网筛,置于棕色样品瓶中,0~4 ℃避光保存,一个月内完成分析。

试验用空白沉积物样品采自湖南省长沙地区的池塘和湖泊。沉积物中的有机质含量采用NY/T 1121.6-2006 《土壤检测 第6部分:土壤有机质的测定》规定的方法进行测定。

### 1.3 样品前处理

称取2 g(精确至±0.02 g)试样于50 mL离心管中,加入4%(体积分数)氨水溶液20 mL,涡旋混合1 min,超声提取10 min,置于水浴恒温振荡器中,于25 ℃振荡提取30 min, 10000 r/min离心5 min。定量吸取2 mL上清液转移过柱,收集流出液,过0.22 μm聚醚砜滤膜,装入聚丙烯进样瓶,供UHPLC-MS/MS测定。

沉积物空白基质溶液制备:称取不含目标物的沉积物样品于离心管中,按照上述步骤进行提取和净化,过0.22 μm滤膜,即可得到空白基质溶液。

### 1.4 标准溶液的配制

取GLUF、GLY及其6种代谢物标准品各约10 mg,精密称定,用水溶解并稀释定容至100 mL,配制成质量浓度均为100 mg/L的标准储备液。分别移取适量上述标准储备液,用水稀释配制成1.0 mg/L和0.1 mg/L的混合标准中间液。移取适量混合标准中间液,用空白基质溶液配制成质量浓度为2.0、4.0、10.0、20.0、50.0、100.0和200.0 μg/L的系列混合标准工作液。

### 1.5 色谱-质谱条件

#### 1.5.1 色谱条件

Metrosep A Supp 5阴离子色谱柱(150 mm×4.0 mm, 5 μm),加装Metrosep A Supp 5 Guard/4.0保护柱(5 mm×4.0 mm, 5 μm)和瑞士万通在线过滤器(2 μm);柱温40 ℃;流速0.6 mL/min;进样量10 μL;流动相A为水,B为200 mmol/L碳酸氢铵溶液(含0. 05%(v/v)氨水);梯度洗脱程序: 0~0.5 min, 10%B; 0.5~1.0 min, 10%B~40%B; 1.0~4.0 min, 40%B~80%B; 4.0~5.0 min, 80%B~82.5%B; 5.0~6.0 min, 82.5%B~95%B; 6.0~12.0 min, 95%B; 12.1 min 10%B; 12.1~15.0 min, 10%B。

#### 1.5.2 质谱条件

电喷雾离子源(ESI),负离子模式,多反应监测(MRM);喷雾电压:-2500 V;离子源温度600 ℃;气帘气(curtain gas)压力为207 kPa,碰撞气(collision gas)压力为62 kPa,喷雾气压力(Gas 1)为310 kPa,辅助加热气(Gas 2)压力为345 kPa。优化后的其他质谱参数见表1。

**表1:** 8种目标化合物的质谱参数

No.	Compound	Compound abbr.	Parent ion (*m/z*)	Product ion (*m/z*)	Declustering potential/V	Collision energy/eV
1	glufosinate	GLUF	180.0	63.0^*^	-60	-68
				85.0		-27
2	3-methyl phosphinicopropionic acid	MPPA	151.0	63.0^*^	-50	-50
				133.0		-19
3	*N*-acetyl glufosinate	*N*-acetyl GLUF	222.0	63.0^*^	-40	-86
				136.0		-27
4	glyphosate	GLY	168.0	63.0^*^	-40	-37
				79.0		-60
5	aminomethyl phosphonic acid	AMPA	110.0	63.0^*^	-60	-26
				79.0		-34
6	*N*-acetyl aminomethyl phosphonic acid	*N*-acetyl AMPA	152.0	63.0^*^	-50	-42
				110.0		-20
7	*N*-acetyl glyphosate	*N*-acetyl GLY	210.0	63.0^*^	-70	-53
				150.0		-19
8	*N*-methyl glyphosate	*N*-methyl GLY	182.0	79.0^*^	-50	-50
				63.0		-83

^*^ Quantitative ion.

## 2 结果与讨论

### 2.1 色谱条件的优化

GLUF、GLY及其代谢物极性强,在常规C_18_色谱柱上难以保留。实验比较了Acquity UPLC BEH Amide(100 mm×2.1 mm, 1.7 μm)、Waters Anionic Polar Pesticide(100 mm×2.1 mm, 5 μm)、Metrosep A Supp 5(150 mm×4.0 mm, 5 μm) 3款适用于强极性化合物分析的色谱柱对目标物的分离效果。结果表明,Acquity UPLC BEH Amide色谱柱对目标物有保留,但色谱峰展宽、拖尾严重,无法对目标物进行准确定量。Waters Anionic Polar Pesticide色谱柱对草铵膦及其代谢物保留较好,色谱峰峰形良好,但对草甘膦及其代谢物分离效果不理想,色谱峰拖尾较严重;Metrosep A Supp 5色谱柱对目标物的分离效果好,色谱峰保留时间适宜,峰形尖锐对称。

Metrosep A Supp 5是一款以带有季铵基团的聚乙烯醇为填料的色谱柱,适用于以离子交换原理分析阴离子型化合物。GLUF、GLY及其代谢物为离子型农药,含有羧基或膦酸基等活性基团,在碱性溶液中主要以阴离子形态存在。实验以水为流动相A,考察了水相中加入不同浓度碳酸氢铵和氨水对色谱峰形及响应值的影响。结果表明,当流动相B为200 mmol/L碳酸氢铵(含0.05%氨水)时,GLUF、GLY及其代谢物在15 min内即可完成色谱分离,色谱峰形良好,响应值高,保留时间稳定。8种目标化合物混合标准溶液(20 μg/L)的MRM色谱图见图1。

**图 1:**
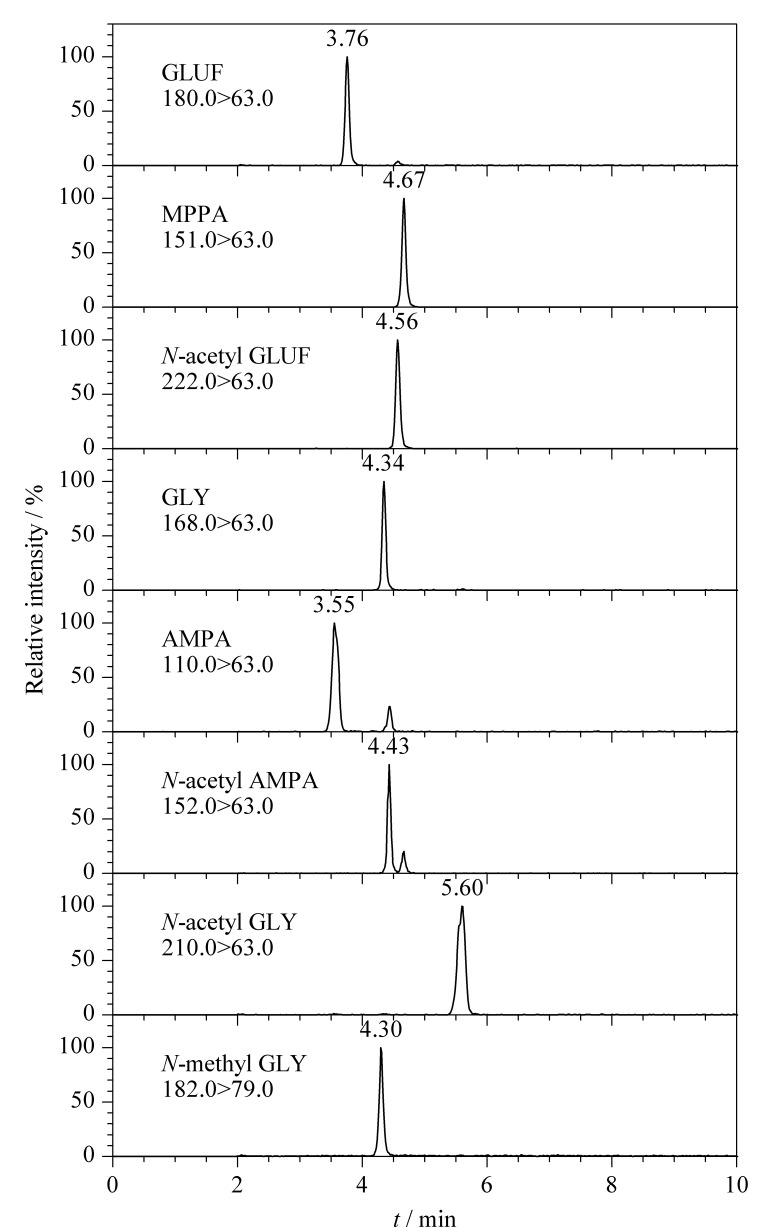
8种目标物(20 μg/L)混合标准溶液的色谱图

### 2.2 质谱条件的优化

将50 μg/L的GLUF、GLY及其代谢物混合标准溶液通过针泵恒流进样方式注入质谱仪中,在负离子模式(ESI^-^)下进行一级质谱扫描(Q1 scan),获得目标化合物的母离子,再使用子离子扫描(product ion scan)模式对获得的母离子进行扫描,确定目标物的子离子。选取2个相对丰度强且稳定的子离子与对应母离子组成定性离子对和定量离子对,采用MRM模式优化选定离子对的去簇电压和碰撞能量,最终确定1.5.2节所述的质谱条件。

### 2.3 前处理条件的优化

#### 2.3.1 提取剂的优化

GLUF、GLY及其代谢物极性强,易溶于水,难溶于大多数有机溶剂。研究表明,该类化合物提取剂一般采用水-二氯甲烷^[5]^、水-甲醇^[15]^、水-乙腈^[16]^、水^[17]^、碱性水溶液^[22]^等,其中采用水和碱性水溶液提取最为常见。本研究选用对质谱友好的氨水溶液作为碱性溶液提取剂,分别比较了水和氨水溶液对目标物提取效率的影响。结果表明,氨水溶液提取时各目标物的回收率均较高,水提取时各目标物回收率均不能令人满意。这可能的原因是GLUF、GLY及其代谢物为离子型农药,主要以离子交换、氢键等作用与沉积物中的有机质、黏土矿物等成分发生吸附,沉积物pH对目标物的吸附量呈负相关,采用碱性溶液作为提取剂时,有利于GLUF、GLY及其代谢物以离子形态从沉积物中解离出来,从而降低沉积物对目标物的吸附,提高回收率^[22,28⇓-30]^。因此,选择氨水溶液作为提取剂。实验进一步考察了不同体积分数(0.5%、1%、2%、4%、8%)的氨水溶液对GLUF、GLY及其代谢物提取回收率的影响(见图2)。结果表明,采用4%和8%的氨水溶液作为提取剂时,8种化合物的回收率均较高(70%~120%),从节省试剂的角度考虑,最终选择4%的氨水溶液作为提取剂。

**图 2:**
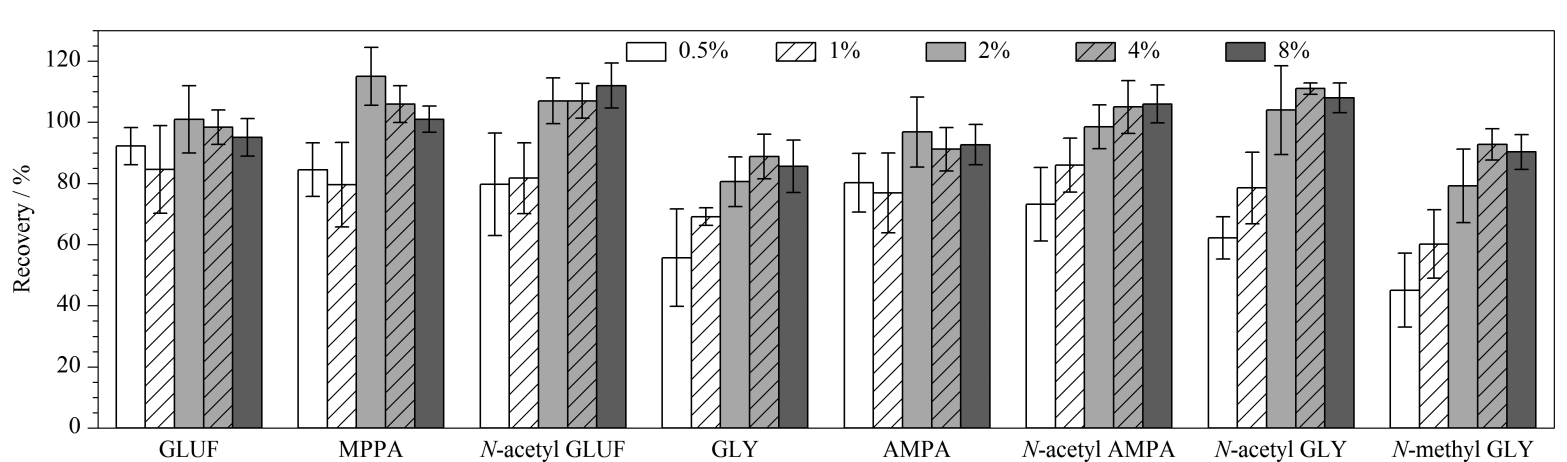
氨水体积分数对GLUF、GLY及其代谢物回收率的影响(*n*=3)

#### 2.3.2 提取温度的优化

温度是可能影响提取回收率的一个因素。本实验考察了5个不同温度(20、30、40、50、60 ℃)对提取回收率的影响(见图3)。结果表明,GLUF、GLY及其代谢物的回收率受温度影响不明显。因此,提取温度设定为室温25 ℃。

**图 3:**
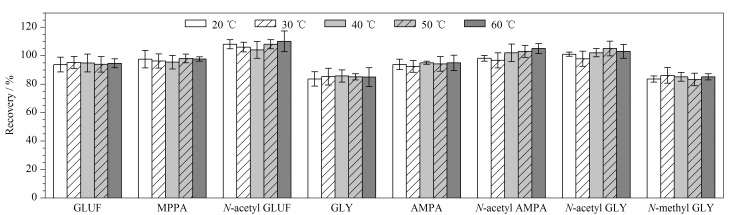
提取温度对GLUF、GLY及其代谢物回收率的影响(*n*=3)

#### 2.3.3 净化条件的优化

GLUF、GLY及其代谢物的净化方式主要有固相萃取法、分散固相萃取法以及不采取净化手段直接检测^[12,22]^。样品不净化虽操作简单快速,但对仪器抗污染能力和外标法准确定量产生巨大挑战,无法应用于大批量样品的准确分析。固相萃取法常将HLB固相萃取柱^[5,21,29]^用于目标物样品前处理净化。分散固相萃取法常采用PSA^[31]^、*C*_18_^[32]^、GCB^[14]^等吸附剂对样品提取液进行净化。本实验考察了3种吸附剂和HLB固相萃取柱对GLUF、GLY及其代谢物回收率的影响。C_18_吸附剂净化后目标物的回收率无明显差别,表明提取液中的疏水性干扰物对目标物检测无显著影响。PSA和GCB吸附剂净化后目标物回收率降低,可能的原因是GLUF、GLY及其代谢物为强极性离子型农药,PSA和GCB吸附剂在吸附色素等极性杂质的同时也对目标物产生了吸附。采用HLB固相萃取柱净化时,目标物的回收率较高,表明其净化效果佳,适用于沉积物样品提取液的净化。但固相萃取前处理过程需经过活化、淋洗、洗脱等步骤,操作繁杂耗时。PRiME HLB柱的填料为水可浸润性的强亲水性聚合物,具有独特的亲水-疏水平衡性,操作时可采用通过式净化模式,提取液直接过柱,小柱无需活化、淋洗和洗脱等步骤,可以去除常见基质干扰物(如磷脂、脂肪、盐和蛋白质),提高回收率。在本实验中,提取液离心后直接过柱,整个净化过程步骤少,操作简单,8种目标物的回收率为77.6%~105%,适用于大批量样品快速检测。因此,本研究最终采用PRiME HLB固相萃取柱净化。

### 2.4 基质效应评价

基质效应(matrix effect, ME)在HPLC-MS/MS分析检测中很常见。ME会影响(增强或抑制)目标物在质谱喷雾接口处的电离,致使分析测定结果的正确度和精密度发生变化^[33]^。本研究采用离子抑制率对ME进行评价,即ME=(*k*_2_/*k*_1_-1)×100%,其中,*k*_1_为溶剂标准曲线的斜率;*k*_2_为空白基质匹配标准曲线的斜率。当|ME|≤20%,表明ME不明显;20%<|ME|≤50%,表明ME中等;|ME|>50%,表明ME很强。结果表明,GLUF、GLY、MPPA、*N*-acetyl GLUF、AMPA、*N*-acetyl AMPA、*N*-acetyl GLY、*N*-methyl GLY基质效应分别为-12.9%、-5.9%、-5.5%、-3.3%、-9.9%、-1.8%、-4.1%、-6.3%,目标物均表现为基质抑制效应,但基质效应不明显。沉积物样品基质较为复杂,在实际测样过程中,本实验采用空白基质匹配标准曲线定量,以确保定量结果的准确性。

### 2.5 方法学验证

#### 2.5.1 线性范围和灵敏度

按照1.4节的方法配制基质匹配标准工作溶液,上机测定,绘制标准曲线。在空白沉积物样品中添加低浓度的标准溶液,按照1.3节的方法处理后测定,以信噪比(*S/N*)=3确定方法的检出限(LOD),以*S/N*=10且回收率为70%~120%确定方法的定量限(LOQ)。GLUF、GLY及其代谢物的线性范围、校准曲线、相关系数(*R*^2^)、检出限和定量限见表2。由表2可知,GLUF、GLY及其代谢物在2.0~200.0 μg/L范围内线性关系良好,*R*^2^均大于0.995。草铵膦、3-甲基磷酸亚基丙酸、*N*-乙酰草铵膦、*N*-乙酰氨甲基膦酸、*N*-乙酰草甘膦、*N*-甲基草甘膦的检出限为5 μg/kg,定量限为20 μg/kg;草甘膦、氨甲基膦酸的检出限为10 μg/kg,定量限为30 μg/kg。本方法灵敏度较高,适用于沉积物中GLUF、GLY及其代谢物的分析测定。

**表2:** GLUF、GLY及其代谢物的线性范围、校准曲线、相关系数、检出限和定量限

Compound	Linear range/(μg/L)	Calibration curve	*R*^2^	LOD/(μg/kg)	LOQ/(μg/kg)
GLUF	2.0-200.0	*y*=2721.544*x*+508.802	0.9988	5	20
MPPA	2.0-200.0	*y*=28554.852*x*+3261.535	0.9992	5	20
*N*-acetyl GLUF	2.0-200.0	*y*=11588.026*x*-135.112	0.9995	5	20
GLY	2.0-200.0	*y*=1208.219*x*+531.743	0.9975	10	30
AMPA	2.0-200.0	*y*=1301.555*x*+1905.21	0.9956	10	30
*N*-acetyl AMPA	2.0-200.0	*y*=5092.339*x*+2308.535	0.9977	5	20
*N*-acetyl GLY	2.0-200.0	*y*=2936.293*x*+468.666	0.9974	5	20
*N*-methyl GLY	2.0-200.0	*y*=2622.722*x*+5109.891	0.9983	5	20

*y*: peak area; *x*: mass concentration, μg/L.

#### 2.5.2 回收率和精密度

沉积物中的有机质对GLUF、GLY及其代谢物产生吸附作用,有机质含量对目标物提取回收率具有重要影响。选取低有机质含量的空白沉积物样品(有机质含量为2.32%)和高有机质含量的空白沉积物样品(有机质含量为7.81%),设定3个加标水平(LOQ、5LOQ、10LOQ),其中GLUF、MPPA、*N*-acetyl GLUF、*N*-acetyl AMPA、*N*-acetyl GLY、*N*-methyl GLY的加标水平分别为20、100、200 μg/kg, GLY、AMPA加标水平分别为30、150、300 μg/kg,每个水平做6个平行样,按本方法进行回收率和精密度试验,结果见表3。

**表3:** 不同有机质含量沉积物中8种目标物的回收率和精密度(*n*=6)

Compound	Spiked/(μg/kg)	Sediment with low organic matter			Sediment with high organic matter		
		Recovery/%	RSD/%		Recovery/%	RSD/%	
GLUF	20	88.2	3.42		82.2		6.23
	100	92.3	5.01		90.5		6.08
	200	96.9	3.04		92.4		4.57
MPPA	20	101	5.28		89.9		4.91
	100	95.6	3.85		103		11.2
	200	89.8	5.20		90.6		2.74
*N*-acetyl GLUF	20	105	2.65		102		3.85
	100	107	4.89		109		4.22
	200	102	2.22		113		8.07
GLY	30	88.8	8.20		76.4		7.62
	150	78.5	6.13		82.5		5.41
	300	83.7	3.92		85.6		4.80
AMPA	30	91.2	7.83		93.5		8.27
	150	93.0	9.79		96.2		4.62
	300	94.8	2.38		90.6		5.15
*N*-acetyl AMPA	20	87.3	1.32		85.5		5.33
	100	93.7	3.80		89.1		2.60
	200	98.3	4.14		96.2		5.23
*N*-acetyl GLY	20	95.7	7.51		101		8.11
	100	101	4.58		97.4		5.24
	200	103	5.02		108		6.07
*N*-methyl GLY	20	85.5	14.7		82.1		8.16
	100	85.3	6.23		79.6		5.02
	200	82.6	4.04		86.1		9.05

由表3可知,低有机质含量沉积物中8种化合物的回收率为78.5%~107%,相对标准偏差为1.32%~14.7%;高有机质含量沉积物中8种化合物的回收率为76.4%~113%,相对标准偏差为2.60%~11.2%,表明该方法的回收率高,重复性好,满足实验要求。

### 2.6 实际样品检测

为了进一步验证方法的适应性,采用本方法对湖南长沙地区的10个池塘沉积物样品(有机质含量为2.14%~5.02%), 5个湖泊沉积物样品(有机质含量为2.56%~6.15%)、5个水库沉积物样品(有机质含量为1.57%~4.51%)以及5个河流沉积物样品(有机质含量为2.38%~5.26%)进行分析检测。结果显示,湖泊、水库、河流沉积物样品中均未检出GLUF、GLY及其代谢物,1个池塘沉积物样品中检出GLY和AMPA,检出含量分别为31.7 μg/kg和52.3 μg/kg。进一步调查发现,该池塘沉积物中检出上述农药的可能原因为池塘位于大型茶园附近,农药在茶园使用后随雨水从土壤和植物体迁移到地表水体中,进而迁移、富集到池塘沉积物中。

## 3 结论

本研究建立了一种非衍生化快速测定沉积物中GLUF、GLY及其代谢物的分析方法。与传统衍生化方法相比,该方法具有简单、快速、绿色环保、灵敏度高、重复性好等优点,可以满足不同有机质含量沉积物中GLUF、GLY及其代谢物的批量快速检测,为研究其在沉积物中的污染状况和环境行为提供了可靠的技术支持。
